# L-Asparaginase-Induced Hepatotoxicity Treated Successfully With L-Carnitine and Vitamin B Infusion

**DOI:** 10.7759/cureus.16917

**Published:** 2021-08-05

**Authors:** Christina Lee, Thomas M Leventhal, Chimaobi M Anugwom

**Affiliations:** 1 Internal Medicine, University of Minnesota Medical School, Minneapolis, USA; 2 Division of Gastroenterology, Hepatology, and Nutrition/Transplant Hepatology and Critical Care Medicine, University of Minnesota, Minneapolis, USA; 3 Gastroenterology and Hepatology, University of Minnesota, Minneapolis, USA

**Keywords:** severe hepatotoxicity, drug-induced hepatotoxicity, cholangiopathy, acute lymphocytic leukemia, asparaginase, l-carnitine, vitamin b

## Abstract

Asparaginase plays an integral role in chemotherapy for acute lymphoblastic leukemia (ALL). We present a 69-year old woman with refractory ALL, who developed asparaginase-induced hepatotoxicity and cholangiopathy after starting intravenous PEG-L-asparaginase-based chemotherapy. The patient was ultimately treated with the combination of L-carnitine and vitamin B complex, resulting in normalization of liver enzymes levels. This case highlights the consideration of PEG-L asparaginase chemotherapy-induced liver steatosis, injury, and cholangiopathy as well as the role of L-carnitine and vitamin B complex as treatment.

## Introduction

Drug-induced liver injury is a complex and diverse syndrome that has been attributed to a variety of therapeutic and non-therapeutic agents [1]. The toxic effect of cancer chemotherapy on the liver ranges from sub-clinical disease to fatal cases of acute liver failure; and management of these toxicities are largely supportive [1]. Here, we describe a case of severe hepatic steatosis and cholangiopathy due to PEG-Asparaginase toxicity, with the resolution of liver injury after discontinuation of PEG-asparaginase use and concomitant treatment with an L-carnitine supplement and vitamin B complex.

## Case presentation

A 69-year-old female was seen in hospital consultation for abdominal pain and altered mental status, after being admitted five days prior for a planned chemotherapy treatment.

She has a medical history of T-cell ALL, diagnosed six months prior to presentation. This diagnosis was made based on biopsies of her submandibular lymph nodes and subsequently, her bone marrow. She had undergone induction chemotherapy with the PETHEMA protocol (vincristine, prednisone, daunorubicin, asparaginase, and cyclophosphamide) two months after diagnosis, followed by Hyper-CVAD therapy (Cyclophosphamide, Vincristine, Doxorubicin, Dexamethasone, Methotrexate, and Cytarabine) for persistent disease after induction. 

A month prior to her hospitalization, a repeat bone marrow biopsy demonstrated low-level but persistent lymphoblastic leukemia. Additional chemotherapies were attempted, but she was unable to tolerate treatment with Vincristine and Nelarabine secondary to adverse side effects, which ultimately lead to the scheduled hospitalization for augmented hyper-CVAD therapy with PEG-asparaginase. 

**Figure 1:**
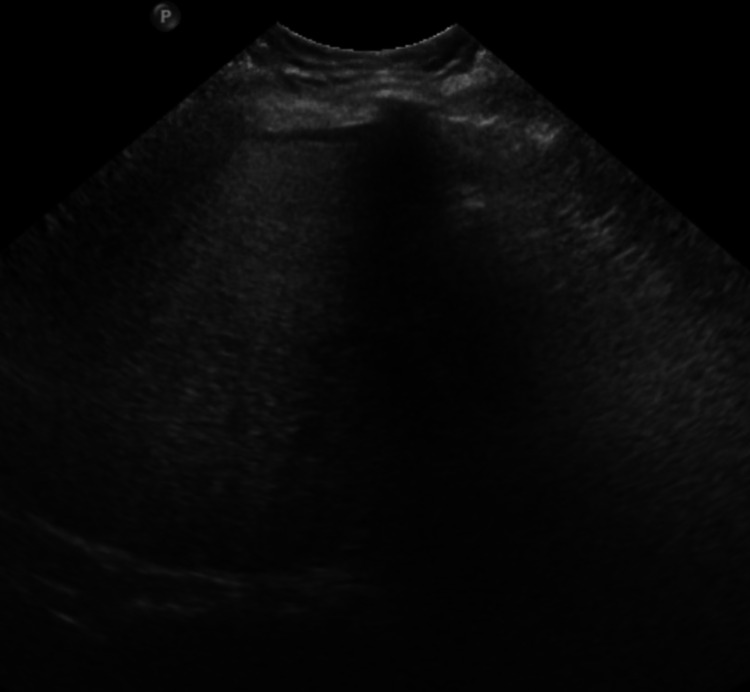
Ultrasound of the liver Abdominal ultrasound showing hepatic steatosis, absence of a gall bladder, and no masses or ascites.

At the time of evaluation, she reported mild nausea but denied vomiting, diarrhea, fever or chills. With ambient air her oxygen saturation was 98%, she was afebrile, but demonstrated tachycardia and tachypnea. Physical examination also elucidated scleral icterus as well as right upper quadrant and epigastric tenderness. Laboratory tests revealed an aspartate aminotransaminase (AST) of 45 U/L, alanine aminotransaminase (ALT) of 72 U/L, alkaline phosphatase (Alk phos) of 282 U/L and total bilirubin (TB) was 2.0 mg/dL. Lipase was elevated at 1687 U/L; greater than three times the upper limit of normal at our laboratory. She was diagnosed with probable drug-induced pancreatitis and drug-induced cholestatic liver injury. An extensive investigation was carried out to determine any other causes of liver injury and pancreatitis. Serological testing revealed that she was immune to Hepatitis B and Hepatitis A, and negative for Hepatitis C infection. An ultrasound of the abdomen revealed hepatic steatosis, absent gall bladder, and absence of ascites (Figure 1).

**Figure 2:**
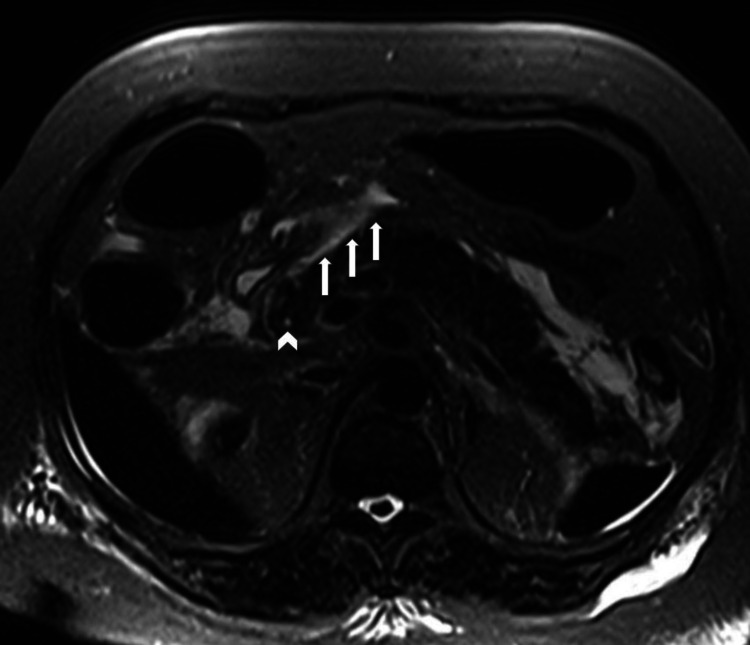
MRCP Magnetic resonance cholangiopancreatography (MRCP) with evidence of edematous pancreatitis (white arrows); the common bile duct as seen here is not dilated (white arrow head) and no other biliary abnormalities were noted.

To further evaluate biliary disease, a magnetic resonance cholangiopancreatography (MRCP) was obtained, which showed interstitial edematous pancreatitis, bilateral pleural effusions, but absent intrahepatic or extrahepatic biliary dilatation or changes concerning for cholangitis (Figure 2).

**Figure 3:**
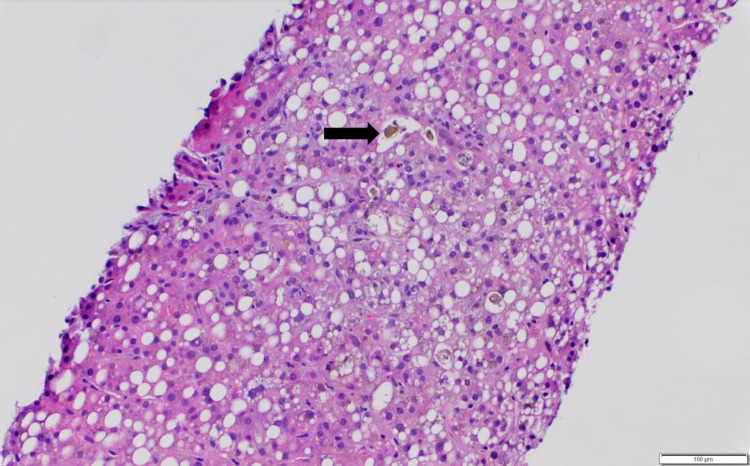
Liver biopsy with hematoxylin and eosin stain (100x) Liver biopsy with hematoxylin and eosin stain showing diffuse steatosis made up of mixed micro and macro-vesicular steatosis, together affecting up to 70%-75% of the parenchyma. Hepato-canalicular cholestasis also shown by the black arrow.

**Figure 4:**
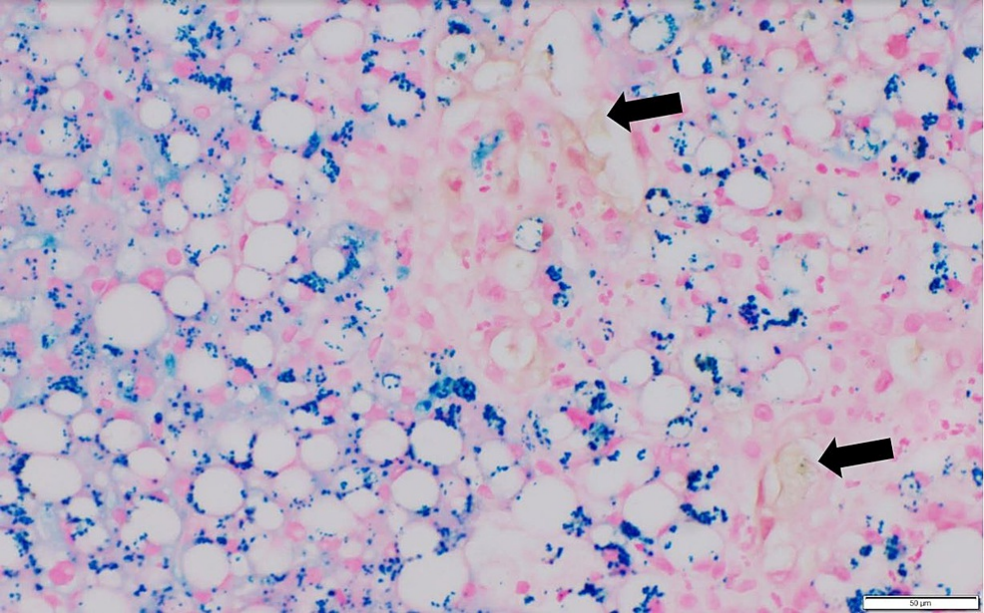
Liver biopsy with Prussian blue staining (400x) Liver biopsy with Prussian blue staining (400x) showing significant hemosiderosis (stained bright blue). Bile pigments (cholestasis) also noted in the specimen (stained brown) and marked by black arrows.

**Figure 5:**
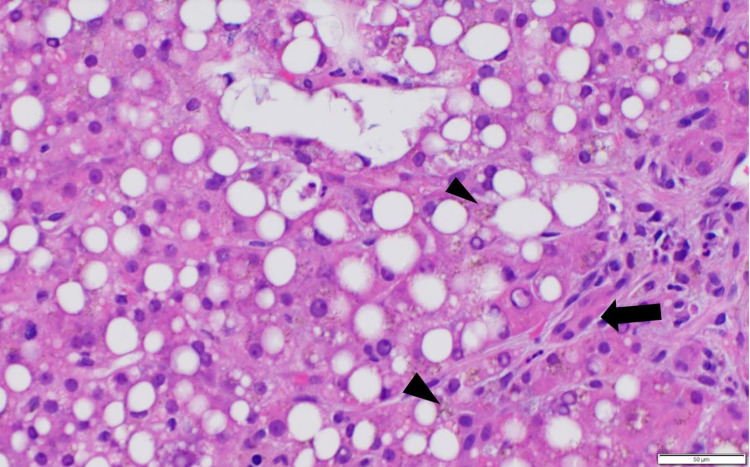
Liver biopsy with hematoxylin and eosin stain (400x) Liver biopsy with hematoxylin and eosin stain (400x) showing diffuse steatosis. Diseased bile duct (cholangiopathy) is demonstrated in this figure with the black arrow. Iron pigments also noted diffusely in the figure, and marked by the black arrowheads.

There was a suspicion of PEG-Asparaginase as the cause of her symptoms and multi-organ injury given the presence of concomitant hepatitis and pancreatitis. Her symptoms of nausea, abdominal pain, and altered mental status improved with a low-fat diet, intravenous fluid resuscitation, and analgesia. She was concomitantly started on L-carnitine supplementation and Vitamin B complex for presumed Asparaginase-induced liver injury. As her liver enzymes and bilirubin continued to increase despite these interventions, a liver biopsy was performed. The pathology revealed severe hepatic steatosis and moderate cholestasis (Figure 3, Figure 4), and mild small duct injury/cholangiopathy (Figure 5); consistent with the limited literature description of histologic characterization of asparaginase hepatotoxicity. 

**Figure 6:**
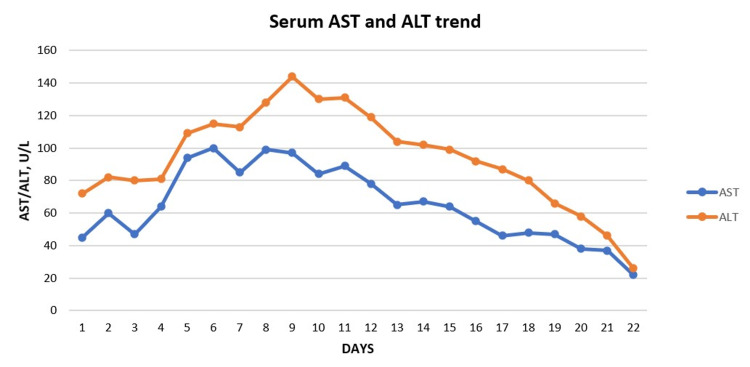
Serum AST and ALT Serum aspartate aminotransferase and alanine aminotransferase are seen decreasing through the course of the patient's hospitalization.

**Figure 7:**
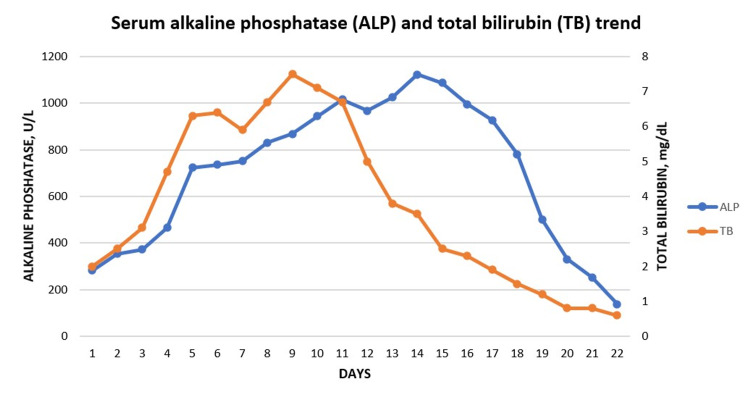
Serum alkaline phosphatase and total bilirubin Serum alkaline phosphatase and total bilirubin are seen decreasing through the course of the patient's hospitalization.

There was no evidence of leukemia, nodular regenerative hyperplasia, granulomas or significant fibrosis. Her liver enzymes, alkaline phosphatase, and bilirubin started to show improvement on day 10 of IV L-carnitine and continued toward normalization thereafter (Figure 6, Figure 7).

Since discharge, the patient has had a bone marrow biopsy without ALL on morphology but 0.06% abnormal lymphoblasts on flow. In order to maintain remission while working on performance status, the patient was started on maintenance chemotherapy with POMP (6-Mercaptopurine, Vincristine, Methotrexate, Prednisone) and she continues to tolerate her current therapy. She is planning to be a part of a clinical trial with CAR-T (chimeric antigen receptor T cell) immunotherapy, once her performance status improves.

## Discussion

Since its introduction to the field of pediatric oncology in the 1960s, Asparaginase has been an integral part of chemotherapy regimens for treating ALL [2, 3]. It is isolated from pathogenic bacteria and is available as L-asparaginase; and PEG-asparaginase - a pegylated form made by adding a mono-methoxypolyethylene moiety [3]. Leukemic cells cannot synthesize L-asparagine and so depend on extracellular sources. Asparaginase catalyzes the breakdown of asparagine to aspartic acid and ammonia, denying the cancer cells of this vital amino acid [4]. It is not inconceivable that depletion of this extracellular amino acid may have deleterious effects on non-malignant human cells.

There is a myriad of adverse effects associated with asparaginase, which include varying severities of hypertriglyceridemia, acute pancreatitis, venous thrombosis, hypersensitivity reactions, neurologic dysfunctions as well as hepatotoxicity and bile duct injury [5-7].

Hepatotoxicity due to asparaginase use may present itself as a self-limiting mild elevation of liver enzymes and bilirubin and in rare cases, severe liver injury with associated death [8,9]. Although the complete mechanism of action is not clear, a putative mechanism is the depletion of asparagine and possibly arginine, with resultant impairment in protein synthesis and lipoprotein export, diminished mitochondrial beta-oxidation of fatty acids, and consequent rapid free fatty acid accumulation [10]. The macroscopic effect of this disorder of protein and lipid metabolism is severe hepatic steatosis, found in up to 90% of patients, with associated biliary injury and cholestasis as was seen in our patient [11]. The biopsy specimen revealed significant micro-vesicular and macro-vesicular steatosis with cholestasis and cholangiopathy.

Spontaneous resolution of hepatitis will occur in most cases of asparaginase-induced liver injury, and treatment is largely supportive. However, the use of L-carnitine has been studied in the treatment of liver toxicity due to asparaginase, carbon tetrachloride, valproic acid, and arsenic [12-14]. The combination with vitamin B complex, as was used in our patient, has been documented to accelerate improvement and resolution of hepatitis, especially in severe cases. It is suggested that by acting as mitochondrial beta-oxidation cofactors, they augment fatty acid oxidation and reduce the severity of steatosis [14-16].

Additionally, our patient had a continued rise in her liver enzymes and bilirubin even after a few days of treatment. This delayed improvement in liver injury has been documented in the literature as well [9].

## Conclusions

We believe that our patient sustained liver injury due to PEG-Asparaginase use, and this improved overtime after administration of L-carnitine and vitamin B complex. It is important to note, however, that the beneficial effects of the combination of L-carnitine and vitamin B complex infusions have not been studied in a randomized controlled trial and that this may be a normal course of L-asparaginase induced hepatotoxicity, and therefore the resolution of liver injury cannot be fully ascribed to these medications and can only be inferred. It is nevertheless plausible to commence this combination treatment regimen in patients with severe asparaginase-induced liver injury.
